# Hormonal Contraception and Vestibular Disorders: A Narrative Review of Clinical Implications, Diagnostic Challenges, and Evidence Gaps

**DOI:** 10.7759/cureus.110393

**Published:** 2026-06-07

**Authors:** Melissa Castillo-Bustamante, Darren M Whelan, Leigh Martin

**Affiliations:** 1 Otoneurology, Centro de Vértigo Medellín, Medellín, COL; 2 Audiology, Interacoustics Academy, London, GBR

**Keywords:** contraceptives, diagnostic medicine, hormonal contraception, hormonal contraceptives, oral contraceptives, sex hormones, use of contraceptives, vestibular disorders

## Abstract

Hormonal influences on inner ear physiology are increasingly recognized, particularly in cochlear function; however, their role in vestibular physiology remains incompletely understood. Hormonal contraceptives represent a major source of exogenous hormonal exposure, yet their effects on vestibular function, symptom expression, and vestibular diagnostic testing remain poorly defined. This review aimed to synthesize current evidence regarding the interactions between hormonal contraceptives and the vestibular system, including biological mechanisms, clinical implications, and diagnostic considerations. A structured narrative review was conducted using PubMed/MEDLINE, Scopus, and Web of Science databases (January 1990-February 2026). Experimental, observational, and translational studies evaluating hormonal effects on vestibular or related neuro-otologic systems were included. Findings were synthesized qualitatively because of methodological heterogeneity and the absence of randomized controlled trials. Available evidence demonstrates the presence of estrogen and progesterone receptors in peripheral and central vestibular structures, supporting the biological plausibility of hormonal modulation. Proposed mechanisms include alterations in ion transport, endolymphatic homeostasis, microvascular perfusion, neurotransmission, and central sensory integration. Clinically, vestibular disorders such as vestibular migraine, Ménière’s disease, and persistent postural-perceptual dizziness show female predominance and symptom fluctuation during hormonal transitions. However, evidence directly linking hormonal contraceptive use with consistent changes in vestibular disease onset, severity, or progression remains limited and heterogeneous. Across vestibular diagnostic modalities, including video head impulse test, vestibular evoked myogenic potentials, videonystagmography, caloric testing, and posturography, no reproducible or clinically significant hormonal effects have been consistently demonstrated in healthy individuals. Current evidence suggests that hormonal contraceptives may act as modulators of vestibular symptoms in susceptible patients, although causality remains unproven. Further prospective vestibular-specific studies are required to clarify their impact on vestibular physiology and clinical disease expression.

## Introduction and background

Hormonal influences on auditory physiology have been increasingly recognized over the past three decades, supported by a growing body of experimental and clinical evidence in cochlear function [1]. In contrast, although analogous mechanisms have been proposed in vestibular physiology, given the shared anatomical structures and fluid continuity within the inner ear, the evidence base remains limited, and key aspects of hormonal modulation of vestibular function are still incompletely understood [1]. Hormonal influences on vestibular physiology are supported by receptor-level findings; however, their clinical relevance remains incompletely characterized. Current evidence is largely derived from mechanistic and observational studies, with limited data from direct human investigations [2].

Accumulating molecular and experimental evidence has demonstrated that both peripheral and central components of the vestibular system are hormonally responsive tissues expressing estrogen and progesterone receptors, including ERα (estrogen receptor alpha) and ERβ (estrogen receptor beta), within the stria vascularis, spiral ligament, endolymphatic sac, vestibular sensory epithelia, and ganglion neurons [3,4]. At the central level, hormone receptors have also been identified in brainstem vestibular nuclei and cerebellar regions involved in sensory integration and vestibulo-ocular reflex modulation [2-5]. These findings support biological plausibility and have contributed to evolving concepts of sex-related differences in vestibular disorders, while also prompting further investigation into the potential influence of exogenous hormonal environments, particularly those induced by hormonal contraceptives, on vestibular homeostasis and disease expression [6].

Hormonal contraceptives are currently used by more than 150 million women worldwide, representing one of the most prevalent long-term pharmacological exposures during reproductive life [7]. Available formulations include combined estrogen-progestin oral contraceptives, progestin-only pills, injectable depot preparations, subdermal implants, transdermal systems, vaginal rings, and levonorgestrel-releasing intrauterine devices [8]. Although the systemic vascular, metabolic, hematologic, and neurological effects of these agents have been extensively studied, particularly in relation to thromboembolic risk and migraine modulation, their potential impact on vestibular physiology remains insufficiently characterized [9,10]. Synthetic estrogens and progestins generate hormonal environments that differ from endogenous ovarian fluctuations, which may plausibly influence ion transport mechanisms, endolymphatic fluid dynamics, microvascular perfusion, and central sensory processing pathways relevant to balance control [11].

Several vestibular disorders demonstrate a marked female predominance, including vestibular migraine, persistent postural-perceptual dizziness (PPPD), Menière’s disease, and motion sensitivity syndromes [12,13]. These conditions often exhibit symptom fluctuation in association with hormonal transitions such as the perimenstrual period, pregnancy, postpartum changes, and perimenopause [12,13]. In vestibular migraine, estrogen withdrawal has been implicated in modulating cortical excitability and trigeminovascular activation, whereas in Menière’s disease, hormonal influences on aquaporin expression, vasopressin sensitivity, and endolymphatic homeostasis have been proposed [14,15]. PPPD, characterized by altered central sensory integration and increased visual dependence, may also be influenced by hormonal modulation of GABAergic tone and limbic network activity [16]. While these observations support biological plausibility, the extent to which exogenous hormonal exposure through contraceptive use influences disease expression or progression remains uncertain [17].

Despite these considerations, clinical guidance regarding contraceptive use in patients with vestibular disorders remains limited and is largely extrapolated from migraine or general otologic literature rather than vestibular-specific studies [18]. Most available evidence derives from observational data, small case series, or indirect mechanistic research, and high-quality prospective studies are lacking [18]. Consequently, clinical decision-making often relies on individualized assessment, particularly in patients with migraine with aura or fluctuating Menière’s disease [19]. Moreover, the potential influence of hormonal contraceptives on vestibular diagnostic testing, including video head impulse test (vHIT), caloric responses, videonystagmography, and vestibular evoked myogenic potentials (VEMP), has not been systematically evaluated [20].

This gap underscores the need for a structured synthesis of the available evidence. The present narrative review aims to integrate current knowledge on the pathophysiological interactions between hormonal contraceptives and the vestibular system, explore disease-specific implications, examine diagnostic and clinical challenges, and identify priorities for future research in this evolving and clinically relevant field [21]. Given the predominantly indirect and non-conclusive nature of the available evidence, this review also seeks to provide practical clinical considerations for patient assessment and highlight key areas where prospective, vestibular-specific research is needed to inform future evidence-based recommendations [21].

## Review

Materials and methods

This study was designed as a structured narrative review aimed at synthesizing current evidence regarding the interaction between hormonal contraceptives and vestibular physiology, as well as their potential influence on specific vestibular disorders and vestibular diagnostic testing outcomes. Given the anticipated heterogeneity of available data and the limited number of studies directly addressing this topic, a narrative synthesis approach was considered methodologically appropriate to integrate mechanistic, clinical, and translational evidence.

A comprehensive literature search was conducted in PubMed/MEDLINE, Scopus, and Web of Science databases covering the period from January 1990 through February 2026. The starting date was selected to capture contemporary research following the molecular characterization of estrogen and progesterone receptors in inner ear tissues and the progressive refinement of vestibular diagnostic methodologies. The search strategy combined controlled vocabulary terms and free-text keywords, including but not limited to the following: “hormonal contraceptives,” “oral contraceptives,” “estrogen,” “progestin,” “progesterone,” “inner ear,” “vestibular system,” “endolymph,” “aquaporins,” “Ménière’s disease,” “vestibular migraine,” “persistent postural-perceptual dizziness,” “dizziness,” “vertigo,” “video head impulse test,” “vHIT,” “videonystagmography,” “VNG,” “caloric testing,” “posturography,” and “vestibular evoked myogenic potentials.”

The database search identified approximately 430 records. After removal of duplicate citations, 318 unique records underwent title and abstract screening. Of these, 96 articles were selected for full-text review. Following the application of the predefined eligibility criteria, 51 references were included in the final synthesis. The included literature comprised observational clinical studies, experimental laboratory investigations, translational studies evaluating hormonal receptor expression and inner-ear physiology, studies examining vestibular function and diagnostic testing, selected case reports, and review articles that provided relevant mechanistic or clinical context. Because the objective of this work was to provide an integrative overview of a relatively underexplored topic rather than to conduct a systematic review, studies were selected based on scientific relevance, methodological quality, and biological plausibility.

Data extraction focused on study design, population characteristics, contraceptive formulation and dosage, duration of exposure, vestibular symptoms or diagnoses, reported changes in vestibular testing parameters, and proposed pathophysiological mechanisms.

Particular attention was given to sample size, participant demographics, duration of contraceptive use, and methodological limitations of studies reporting clinical vestibular manifestations. Given the methodological heterogeneity of the included literature and the absence of randomized controlled trials specifically evaluating vestibular outcomes in contraceptive users, no formal meta-analysis was performed. Instead, findings were synthesized qualitatively, emphasizing mechanistic plausibility, clinical coherence, and areas of concordance or controversy across studies.

Exclusion criteria included non-peer-reviewed publications, editorials without scientific data, purely obstetric or gynecological studies lacking neuro-otologic relevance, and studies focusing exclusively on auditory outcomes without mechanistic implications for vestibular physiology. Articles not available in English were excluded unless a reliable English abstract provided sufficient methodological and results information for interpretation.

To avoid overinterpretation of experimental findings, mechanistic evidence derived from animal models, cellular studies, receptor-expression analyses, and translational investigations was evaluated separately from clinical evidence whenever possible. Consequently, proposed mechanisms involving ion transport regulation, aquaporin expression, endolymphatic homeostasis, neurovascular modulation, and central sensory processing are presented as biologically plausible hypotheses supported by experimental data but not necessarily confirmed clinical effects in hormonal contraceptive users.

Physiopathological mechanisms linking hormonal contraceptives and vestibular function

Hormone Receptors in the Inner Ear

The identification of estrogen and progesterone receptors within the inner ear has expanded current conceptual models of vestibular physiology [2,4]. Immunohistochemical and molecular studies have demonstrated the presence of estrogen receptor subtypes ERα and ERβ, as well as progesterone receptors, in multiple cochleo-vestibular structures, including the stria vascularis, spiral ligament, endolymphatic sac, vestibular sensory epithelia of the semicircular canals and otolith organs, Scarpa’s ganglion, and cochlear ganglion neurons [22]. This widespread receptor distribution suggests that sex steroid hormones may play a regulatory role in inner ear function. However, while these findings support biological plausibility, direct functional consequences in humans have not been clearly established [2,4].

Within the stria vascularis, estrogen receptors are strategically positioned to influence ion transport and endocochlear potential generation [22,23]. In the spiral ligament, fibrocytes involved in potassium recycling express hormonal receptors, supporting the hypothesis that sex steroids may regulate ion homeostasis [22,23]. The endolymphatic sac, a key structure in endolymph volume regulation and immunologic surveillance, also exhibits receptor expression, implicating hormonal signaling in fluid resorption mechanisms [22,23]. Vestibular hair cells and supporting cells similarly demonstrate hormonal responsiveness, suggesting that peripheral sensory transduction itself may be hormonally modulated [22,23].

Exogenous hormones delivered through hormonal contraceptives bind to these receptors under pharmacological conditions that differ from endogenous ovarian cycling [24]. Combined oral contraceptives typically maintain relatively stable circulating estrogen and progestin levels with periodic withdrawal phases, whereas progestin-only methods create selective receptor stimulation without significant estrogenic activity [24]. This continuous or selective receptor engagement may alter baseline gene transcription, ion channel expression, and fluid regulation within the inner ear [24-26]. The sustained activation of estrogen receptors may theoretically influence aquaporin expression, Na⁺/K⁺-ATPase activity, and microvascular permeability, thereby affecting the delicate equilibrium required for vestibular function [24]. However, no published clinical studies have demonstrated hormone-driven alterations in endolymphatic volume, hydrops, or vestibular dysfunction in humans attributable to these mechanisms [4].

The potential vestibular implications of progestin-only contraceptive methods deserve particular consideration given their increasing use in women for whom estrogen-containing contraceptives are contraindicated, especially those with migraine with aura and elevated vascular risk profiles [18,25]. These formulations include progestin-only pills, levonorgestrel-releasing intrauterine devices, subdermal implants, and depot medroxyprogesterone acetate injections. Unlike combined hormonal contraceptives, these methods do not expose users to exogenous estrogen and therefore avoid estrogen withdrawal fluctuations and estrogen-mediated vascular effects that have been implicated in migraine pathophysiology [14,18,26-29]. Nevertheless, progesterone receptors are widely distributed throughout cochleo-vestibular tissues [3,22], suggesting that progestins may still influence inner-ear physiology through receptor-mediated mechanisms. Experimental and neuroendocrine studies indicate that progestins can modulate neuronal excitability, GABAergic neurotransmission, fluid regulation pathways, and components of the renin-angiotensin-aldosterone system, all of which may theoretically influence vestibular processing and homeostasis [21,26]. However, direct clinical evidence evaluating vestibular symptoms, vestibular disorders, or vestibular testing outcomes among users of progestin-only contraceptives remains extremely limited. Consequently, any vestibular effects associated with these formulations should currently be regarded as biologically plausible but clinically unconfirmed.

Importantly, receptor polymorphisms and individual variability in receptor density may partially explain why only a subset of women experience vestibular symptom modulation under hormonal contraceptive exposure [24]. The heterogeneity observed clinically likely reflects differences in receptor sensitivity, downstream signaling pathways, and preexisting vestibular vulnerability [24].

Ion Homeostasis and Endolymphatic Regulation

The maintenance of endolymphatic homeostasis is central to normal vestibular and auditory function [24]. Endolymph is characterized by high potassium and low sodium concentration, a gradient maintained by Na⁺/K⁺-ATPase pumps, potassium recycling mechanisms through supporting cells and fibrocytes, and aquaporin-mediated water transport [24]. Small perturbations in this tightly regulated environment may significantly alter hair cell transduction and mechanoelectrical sensitivity [24,25].

Estrogen has been shown to modulate Na⁺/K⁺-ATPase expression and activity in epithelial tissues, and similar mechanisms are proposed within the stria vascularis and vestibular dark cells [24,25]. Furthermore, estrogen influences aquaporin-2 and aquaporin-4 expression, proteins that regulate water permeability and osmotic balance [24,25]. Upregulation of aquaporins under sustained estrogen exposure may theoretically increase endolymphatic fluid accumulation in susceptible individuals [24,25].

Estrogen modulates aquaporins in experimental models; however, increased endolymphatic volume in contraceptive users has not been demonstrated in clinical research [4,23,24]. In the endolymphatic sac, estrogen-mediated changes in epithelial transport could influence hydrostatic pressure and resorptive capacity [4,24,25].

Progesterone and synthetic progestins exert distinct but complementary effects [26]. Systemically, progestins may promote sodium retention and modify renin-angiotensin-aldosterone system activity [26]. They may also alter vasopressin sensitivity and microvascular permeability [26]. Within the inner ear, enhanced vasopressin signaling has been implicated in the development of endolymphatic hydrops [26]. Therefore, progestin-induced modulation of vasopressin pathways could influence inner ear fluid dynamics, particularly in patients predisposed to hydrops [26]. Progestins can affect systemic sodium/vasopressin pathways, though vestibular effects remain hypothetical, with no human evidence of measurable endolymphatic changes.

Sustained estrogen exposure combined with progestin-mediated fluid retention may create a hormonal milieu conducive to subtle increases in endolymphatic hydrostatic pressure [27]. While this effect may be clinically silent in most individuals, patients with pre-existing hydropic susceptibility, such as those with Menière’s disease, may experience symptom exacerbation [27]. It remains uncertain whether hormonal contraceptives directly induce hydrops or simply amplify an underlying vulnerability; however, mechanistic plausibility is strong [27].

Vascular and Neurovascular Effects

The vestibular labyrinth is highly dependent on stable microvascular perfusion [28]. Estrogen enhances nitric oxide synthase activity, increasing nitric oxide production and promoting vasodilation [28]. Under physiological conditions, this may improve cerebral and labyrinthine blood flow [28]. However, synthetic estrogen used in contraceptives may exert additional effects on coagulation pathways, increasing hepatic synthesis of clotting factors, altering fibrinolytic balance, and modifying platelet aggregation [28].

These prothrombotic effects are well documented in the context of stroke risk in women with migraine with aura using combined hormonal contraceptives [28,29]. Vestibular migraine shares pathophysiological mechanisms with migraine with aura, including cortical spreading depolarization, trigeminovascular activation, and neurogenic inflammation [29]. It is therefore plausible that estrogen-containing contraceptives could exacerbate neurovascular instability in vestibular migraine patients [29].

Microvascular instability may also influence inner ear perfusion [30]. The labyrinthine artery is an end artery with limited collateral circulation, rendering the inner ear vulnerable to ischemic insults [30]. Although overt ischemic vestibulopathy is rare in contraceptive users, subclinical perfusion fluctuations could potentially modulate symptom thresholds in migraine-prone individuals [30]. Whether hormonal contraceptives directly alter labyrinthine blood flow in a clinically significant manner remains to be established [30]. Similarly, rare phenomena such as hormonally mediated extragenital bleeding (vicarious menstruation) have been described in other tissues, further supporting the concept that sex hormones may influence microvascular behavior beyond reproductive organs; however, no evidence currently links this phenomenon to vestibular dysfunction or inner-ear perfusion abnormalities [28-30]. Importantly, the vascular and neurovascular effects associated with hormonal contraceptive use should be interpreted within the broader context of an individual's cardiovascular risk profile. Comorbidities such as hypertension, obesity, smoking, insulin resistance, dyslipidemia, and other prothrombotic conditions may independently contribute to endothelial dysfunction, altered vascular reactivity, impaired microcirculatory regulation, and increased thrombotic risk [28,29]. These factors may interact with hormonal exposure, potentially amplifying susceptibility to migraine-related neurovascular phenomena and theoretically influencing labyrinthine perfusion. Consequently, any vestibular manifestations occurring during hormonal contraceptive use are likely multifactorial and may reflect the combined influence of hormonal, vascular, metabolic, and patient-specific risk factors rather than a direct effect of contraceptive therapy alone [28-30].

Central Vestibular Modulation

Beyond peripheral effects, hormonal contraceptives may influence central vestibular processing [30]. Estrogen modulates glutamatergic transmission, enhances NMDA (N-methyl-D-aspartate) receptor expression, and influences synaptic plasticity [31]. It also affects GABAergic inhibition, altering neuronal excitability within cortical and brainstem networks [31]. Progesterone and its neuroactive metabolites, such as allopregnanolone, exert GABA_A receptor-mediated inhibitory effects, potentially influencing vestibular compensation and anxiety circuits [31].

Estrogen withdrawal is particularly relevant in migraine physiology [31]. Abrupt declines in estrogen levels lower cortical excitability thresholds and facilitate trigeminovascular activation [31]. During the hormone-free interval of cyclic contraceptives, this withdrawal phenomenon may precipitate vestibular migraine episodes, motion intolerance, or increased sensory hypersensitivity [31]. Conversely, continuous low-dose estrogen regimens may attenuate withdrawal-related fluctuations [31].

The vestibular nuclei, cerebellum, thalamus, and parieto-insular vestibular cortex are all involved in multisensory integration and spatial orientation [31]. Hormonal modulation of synaptic plasticity within these structures may influence adaptation processes following vestibular injury or during chronic dizziness states [12]. Hormonal variability could theoretically impair central compensation in patients recovering from acute vestibular neuritis or exacerbate maladaptive sensory weighting in PPPD [11].

Clinical manifestations associated with hormonal contraceptives

Clinical reports indicate that a subset of women experience vestibular symptoms temporally associated with hormonal contraceptive use [32]. These symptoms range from intermittent dizziness and light-headedness to motion sensitivity, transient imbalance, and exacerbation of pre-existing migraine-related vertigo [32]. In patients with established Menière’s disease, increased frequency or severity of vertigo attacks has been reported following initiation of combined hormonal contraceptives [32].

Symptom onset may correlate with hormonal transitions in select individuals; however, these patterns are inconsistent and not supported by controlled studies [32]. A working hypothesis could be that the initiation of contraceptive therapy may produce transient disequilibrium as the central nervous system adapts to altered hormonal signaling [32]. Switching between formulations with different estrogen doses or progestin types may similarly provoke symptom fluctuation [32]. The hormone-free interval of cyclic regimens represents a particularly vulnerable period, during which estrogen withdrawal may trigger migraine-related vestibular episodes [14,32]. Similar symptom patterns have been observed in menstrual-related conditions and during the premenstrual phase, when fluctuations in endogenous sex hormones may contribute to dizziness, light-headedness, impaired balance, headache, motion sensitivity, and worsening of vestibular migraine symptoms in susceptible individuals [14,33]. Premenstrual symptom complexes are frequently characterized by neurovegetative and sensory complaints, suggesting that hormonal fluctuations may influence central sensory processing and symptom perception beyond the reproductive system [14]. These observations support the hypothesis that abrupt hormonal transitions, rather than absolute hormone concentrations alone, may be important determinants of vestibular symptom expression.

Importantly, the clinical pattern frequently reflects abrupt hormonal changes rather than steady exposure alone [32]. Continuous regimens that eliminate withdrawal phases may reduce symptom variability in some patients, whereas cyclic regimens may perpetuate predictable monthly exacerbations [14,32]. Postpartum resumption of hormonal contraception may also coincide with destabilization in hormonally sensitive individuals [32]. Nevertheless, considerable interindividual variability exists, and not all women experience symptom modulation under contraceptive use [32]. The overlap between vestibular symptoms, menstrual migraine, premenstrual symptom exacerbations, and hormonally mediated worsening of vestibular disorders further suggests that individual susceptibility, underlying vestibular disease, and neuroendocrine responsiveness likely play important roles in determining clinical manifestations [14,33].

Disease-specific considerations

Menière’s Disease

Menière’s disease exhibits a recognized female predominance and frequently demonstrates perimenstrual exacerbations, suggesting hormonal influence on endolymphatic regulation [33]. The pathophysiology of Menière’s disease involves endolymphatic hydrops, characterized by expansion of the endolymphatic compartment and mechanical distortion of sensory structures [34]. Hormonal modulation of aquaporin expression, vasopressin sensitivity, and sodium-water balance may theoretically influence hydrops severity [34].

Estrogen-induced upregulation of aquaporins could enhance water permeability in the endolymphatic sac, while progesterone-mediated fluid retention may augment systemic and local hydrostatic pressure [34]. Altered vasopressin signaling may further impair endolymph resorption [34]. These mechanisms suggest that hormonal contraceptives, particularly combined estrogen-progestin formulations, could exacerbate hydropic tendencies in susceptible individuals [34].

Hormonal mechanisms may plausibly interact with hydropic physiology; however, no clinical trials or cohort studies have demonstrated that hormonal contraceptives worsen Ménière’s disease [2]. Although estrogen and progesterone may influence fluid regulation and inner ear homeostasis, current clinical evidence remains limited and largely indirect [2].

Clinical observations are heterogeneous [35]. Some patients report changes in symptom frequency after initiating combined hormonal contraceptives, whereas others describe no clear effect [2]. Additionally, some individuals anecdotally report symptom stabilization with continuous regimens that minimize estrogen withdrawal; however, these observations are supported only by limited clinical data and have not been systematically evaluated in controlled vestibular studies [2].

Overall, available evidence does not support a consistent or clinically predictable effect of hormonal contraceptives on Ménière’s disease activity [35]. These findings underscore the need for cautious interpretation of patient-reported associations and highlight the importance of individualized clinical assessment rather than assuming a direct causal relationship [2].

Vestibular Migraine

Vestibular migraine is strongly influenced by hormonal fluctuations, particularly estrogen withdrawal [36]. Estrogen levels modulate the neurovascular and neuroinflammatory cascades underlying migraine, and abrupt declines in estrogen may precipitate attacks [36].

Combined hormonal contraceptives may influence vestibular migraine through estrogen withdrawal mechanisms, depending on formulation and dosing patterns [37]. Importantly, their use is contraindicated in individuals with migraine with aura due to a well-established increased risk of ischemic stroke, particularly with estrogen-containing formulations [37]. This association highlights the need for careful risk stratification and alternative contraceptive strategies in this population [37].

Continuous low-dose estrogen regimens may stabilize hormonal fluctuations and reduce attack frequency in some patients, whereas cyclic regimens with hormone-free intervals may perpetuate withdrawal-triggered episodes [36]. In women with migraine with aura, combined estrogen-containing contraceptives increase ischemic stroke risk, necessitating careful risk stratification [36].

Progestin-only methods are generally considered safer in individuals with migraine with aura; however, evidence regarding their specific impact on vestibular symptoms is limited. Although they may be associated with a lower risk of migraine exacerbations, their role in modulating vestibular manifestations remains unclear [35].

Persistent Postural-Perceptual Dizziness (PPPD)

PPPD is characterized by chronic non-spinning dizziness, postural instability, visual dependence, and altered multisensory integration [38]. The disorder predominantly affects women and commonly emerges following an acute vestibular event, vestibular migraine, medical illness, or periods of psychological stress [38]. Contemporary models propose that PPPD results from maladaptive shifts in sensory weighting, increased reliance on visual cues, heightened self-motion monitoring, and persistent activation of threat-detection networks [16,38].

Several mechanisms suggest a potential, although currently unproven, interaction between sex hormones and PPPD symptom expression. Estrogen and progesterone influence multiple central nervous system pathways involved in sensory integration, emotional regulation, and autonomic control. Experimental and neuroimaging studies have demonstrated hormonal effects on limbic structures, including the amygdala, hippocampus, anterior cingulate cortex, and insular networks, regions that have also been implicated in PPPD pathophysiology [16,38]. In addition, estrogen and progesterone metabolites modulate GABAergic neurotransmission and cortical excitability, potentially influencing the balance between vestibular, visual, and somatosensory inputs that is often disrupted in PPPD [21,38].

Hormonal fluctuations may also interact indirectly with PPPD through their effects on anxiety, stress responsiveness, and autonomic regulation. Women with menstrual-related migraine, premenstrual symptom exacerbations, or heightened hormonal sensitivity frequently report fluctuations in symptoms associated with hormonal transitions [14,38]. Given the recognized association between PPPD, anxiety-related traits, hypervigilance, and autonomic dysregulation, it is biologically plausible that abrupt hormonal changes could influence symptom severity in susceptible individuals [38]. However, this hypothesis remains speculative and has not been specifically investigated in PPPD populations.

Despite these theoretical mechanisms, empirical data directly evaluating the effects of hormonal contraceptives on PPPD are currently lacking [38]. No studies have assessed whether contraceptive initiation, discontinuation, formulation changes, or hormone-withdrawal intervals influence PPPD symptom severity, visual dependence, postural control, or functional outcomes. Therefore, any proposed interaction between hormonal contraceptives and PPPD should be regarded as biologically plausible but clinically unconfirmed. Future studies examining hormonal status, contraceptive exposure, autonomic function, and symptom trajectories in PPPD populations may help clarify whether hormonal factors contribute to chronic dizziness susceptibility or persistence [16,38].

Impact on vestibular diagnostic testing

An important and often overlooked consideration in the interpretation of vestibular diagnostic testing is the potential influence of endogenous hormonal fluctuations and exogenous hormonal exposure on test parameters [12]. Understanding vestibular test results within the broader context of menstrual cycle-related hormonal variability is essential for accurate differential diagnosis of vestibular disorders in women of reproductive age [12]. While hormonal modulation of inner ear physiology is well established at the molecular level, the extent to which these fluctuations translate into measurable changes in vestibular function remains controversial [12].

Video Head Impulse Test (vHIT)

The vHIT evaluates the high-frequency vestibulo-ocular reflex (VOR) and provides quantitative measurements of semicircular canal gain and corrective saccade patterns [39]. From a mechanistic perspective, hormonal fluctuations could influence VOR dynamics through modulation of hair cell transduction, synaptic transmission in Scarpa’s ganglion, or central vestibular nucleus excitability [39]. Estrogen-mediated effects on ion transport and synaptic plasticity, as well as progesterone-related modulation of GABAergic inhibition, further support the biological plausibility that hormonal variability may influence vestibular function [39,40].

Despite these theoretical considerations, empirical evidence consistently indicates that VOR gain remains stable across menstrual cycle phases in healthy individuals, with no significant differences observed between follicular, ovulatory, and luteal stages [41]. Furthermore, current data do not support a clinically meaningful effect of hormonal contraceptive use on vHIT-derived parameters [41]. These findings suggest that physiological hormonal fluctuations do not significantly alter high-frequency semicircular canal function as assessed by vHIT [41].

From a clinical standpoint, minor variations in gain or corrective saccade patterns should therefore be interpreted with caution and not readily attributed to hormonal influences alone [42]. In patients with vestibular migraine, transient central modulation, particularly during estrogen withdrawal, may theoretically influence corrective saccade generation or subtle gain variability; however, such changes are more likely to reflect central sensitization rather than primary peripheral dysfunction [42]. Consequently, abnormal vHIT findings in individuals using hormonal contraceptives should prompt thorough evaluation for underlying vestibulopathy rather than being presumed to represent hormonal artifacts [42].

Vestibular Evoked Myogenic Potentials (VEMP)

The expression of estrogen and progesterone receptors within inner ear structures supports the hypothesis that hormonal states may influence vestibular as well as auditory function [43]. Given the anatomical proximity and fluid continuity between the cochlea and vestibular labyrinth, along with documented hormonal effects on cochlear physiology, it is biologically plausible that hormonal fluctuations could also impact otolith organ function [43].

However, findings regarding vestibular responses across the menstrual cycle remain equivocal [43]. Studies evaluating cervical VEMP (cVEMP) and ocular VEMP (oVEMP) responses in healthy women have not demonstrated significant differences in latency or amplitude parameters across menstrual phases [43]. These results suggest that physiological hormonal oscillations do not produce measurable alterations in otolith-mediated vestibular function under normal conditions [43].

Although hormonal influences may still play a role in specific clinical populations or under non-physiological conditions (for example, hormonal therapy or endocrine disorders), current evidence does not support a consistent or clinically relevant effect of hormonal fluctuations on VEMP parameters [43]. Overall, while the biological plausibility is well established, the functional impact of hormonal variability on otolith responses appears limited and not reliably reproducible [43].

Videonystagmography (VNG)

Videonystagmography (VNG) evaluates spontaneous, positional, and caloric-induced nystagmus, as well as oculomotor function, providing a comprehensive assessment of both peripheral and central vestibular pathways [44,45]. Hormonal influences on VNG parameters remain incompletely characterized [45]. While some studies have reported menstrual cycle-related variations, such as increased directional preponderance or subtle caloric asymmetries, these findings are inconsistent and not reproducible across studies [45].

Clinical manifestations potentially related to hormonal fluctuations, including giddiness, nausea, vomiting, and headache, appear to occur only in a minority of women, with reported prevalence ranging from 5% to 20% [45]. Experimental data suggest that hormonal changes may influence certain aspects of postural control, such as lateral sway, without affecting anterior-posterior stability or central vestibular responses, including optokinetic function [45]. These findings support the notion that observed variations are likely multifactorial and not solely attributable to vestibular mechanisms [45].

In patients with vestibular migraine, estrogen withdrawal may contribute to central sensitization, potentially increasing susceptibility to spontaneous or positional nystagmus [45]. However, such effects are more consistent with migraine-related central mechanisms rather than direct hormonal modulation of peripheral vestibular function [45].

Caloric Testing

Most observed fluctuations in caloric responses are better explained by disease activity, migraine-related central modulation, or methodological variability rather than by hormonal contraceptive use per se [45,46]. At present, no high-quality evidence demonstrates that hormonal contraceptives directly alter caloric test outcomes in a predictable or clinically relevant manner [45,46].

Although estrogen-mediated changes in vascular tone and fluid regulation could theoretically influence caloric convection dynamics or nystagmus intensity, controlled studies have not established consistent hormonal phase-dependent alterations in caloric responses among healthy women [45,46]. Premenstrual fluid retention may transiently affect endolymphatic volume and peripheral responsiveness in susceptible individuals, but these effects remain variable and not universally observed [45,46].

Some studies have reported menstrual cycle-related changes in vestibular parameters, including saccadic eye movements, directional preponderance, and caloric asymmetries [45,46]. However, these findings are inconsistent and likely reflect individual susceptibility rather than a uniform physiological effect [45,46]. Overall, current evidence suggests that hormonal influences on caloric responses are heterogeneous, limited, and clinically non-predictable [45,46].

Computerized Posturography

Computerized posturography provides a quantitative assessment of postural control through the integration of visual, vestibular, and somatosensory inputs and is commonly used to evaluate sensory organization and balance strategies. From a mechanistic perspective, hormonal fluctuations could plausibly influence postural stability via modulation of central sensory integration, vestibulospinal pathways, and neuromuscular control [47]. The presence of estrogen and progesterone receptors in central nervous system regions involved in balance regulation further supports this hypothesis [47].

However, available evidence indicates that physiological hormonal fluctuations across the menstrual cycle do not result in consistent or clinically significant changes in posturographic parameters in healthy individuals [48]. Reported variations in equilibrium scores or sensory organization test (SOT) conditions are inconsistent and likely reflect interindividual variability or methodological heterogeneity rather than a reproducible hormonal effect [48].

In clinical populations, particularly in individuals with vestibular migraine or chronic vestibular disorders, hormonal changes may contribute to transient alterations in postural control, potentially mediated by central mechanisms such as sensory reweighting or altered multisensory integration [49]. Nevertheless, there is currently no high-quality evidence demonstrating that hormonal contraceptive use produces systematic or clinically meaningful changes in posturography outcomes [49]. Overall, the influence of hormonal factors on postural control appears to be subtle, variable, and not reliably reproducible, and should not be considered a primary determinant of posturographic findings in clinical practice [49].

Diagnostic challenges and controversies

Hormonal contraceptive use is often insufficiently explored during vestibular history-taking [50]. Failure to systematically assess contraceptive type, dosing pattern, and their temporal relationship with symptom onset may contribute to the misattribution of dizziness to anxiety disorders or nonspecific causes [50]. Although the effects of hormonal contraceptives on vestibular symptoms remain poorly characterized, incorporating a structured contraceptive history can help contextualize symptom onset and reduce diagnostic errors [50]. Importantly, hormonal contraceptives should be regarded as potential modifiers rather than established causes of dizziness; however, their role should be considered in the differential diagnosis to avoid misinterpretation of symptoms [50]. Failure to do so may not only lead to diagnostic inaccuracies but also contribute to the overuse of neuroimaging and the underrecognition of hormonally modulated triggers [51]. Dizziness associated with hormonal changes (hereafter referred to as hormone-related dizziness) may mimic PPPD, chronic subjective dizziness, vestibular migraine, or fluctuating endolymphatic hydrops [14,51].

Temporal pattern recognition remains the most valuable diagnostic tool [40]. Detailed symptom diaries aligned with contraceptive cycles can clarify causal relationships and guide management decisions [50]. The role of estrogen in vestibular physiology remains paradoxical [14]. Stable estrogen levels may reduce migraine variability in some patients, whereas withdrawal clearly increases attack risk [14]. Continuous exposure may theoretically worsen hydrops in predisposed individuals. Individual susceptibility, receptor polymorphisms, and disease-specific mechanisms likely determine whether estrogen exerts protective or harmful effects [14].

Future investigations should include prospective cohort studies correlating contraceptive formulation with vestibular symptom diaries, longitudinal evaluation of vHIT gain variability across hormonal cycles, experimental studies examining aquaporin expression under synthetic hormone exposure, and controlled trials comparing continuous versus cyclic regimens in vestibular migraine populations [20]. Mechanistic imaging studies exploring hormonal modulation of vestibular cortical networks may further elucidate central effects [20]. A practical approach for clinicians evaluating patients with dizziness during hormonal contraceptive use is summarized in Table 1.

**Table 1:** Practical clinical approach for the evaluation of hormonal contraceptive use in patients with dizziness and vestibular symptoms.

Clinical recommendation	Practical application
Document contraceptive type	Combined vs. progestin-only formulation
Record duration of use	Date of initiation, discontinuation, or switching
Assess the temporal relationship	Correlate symptom onset with contraceptive changes
Evaluate hormone-free intervals	Identify symptoms during withdrawal periods
Consider menstrual patterns	Assess symptom fluctuation across the menstrual cycle
Use symptom diaries	Track dizziness, migraine, imbalance, and vertigo episodes
Review comorbidities	Migraine, anxiety, hypertension, obesity, and vascular risk factors

## Conclusions

Hormonal contraceptives exert biologically plausible effects on the vestibular system and may influence symptom expression in susceptible individuals; however, current evidence remains limited and insufficient to establish causality. Proposed mechanisms include modulation of endolymphatic homeostasis, neurovascular regulation, and central sensory processing. While physiological hormonal fluctuations do not consistently alter objective vestibular test parameters, exogenous hormones may affect symptoms, particularly in vestibular migraine and Menière’s disease. Clinically, systematic inquiry into contraceptive use, contraceptive formulation, duration of exposure, and temporal correlation with symptom fluctuations should be incorporated into the assessment of women presenting with dizziness or vestibular complaints. Hormonal modulation should be considered a potential contributing factor rather than a primary cause, particularly in patients with vestibular migraine, hormonally sensitive symptom patterns, or fluctuating vestibular disorders.

Given the current limitations of the evidence, individualized clinical decision-making remains essential. Future research should prioritize prospective vestibular-specific studies evaluating different contraceptive formulations, symptom trajectories, vestibular testing outcomes, and relevant hormonal transitions. Longitudinal studies integrating symptom diaries, hormonal profiles, and objective vestibular assessments may help clarify causality and identify susceptible patient subgroups. Such investigations are necessary to support evidence-based recommendations and improve the clinical management of women with vestibular disorders.
